# On the Etiology, Pathology and Treatment of Spermatorrhœa and Impotence

**Published:** 1877-11

**Authors:** 


On the Etiology, Pathology and Treatment of Spermatorrhœa and Impotence.—The subject to which I desire to call your attention this afternoon is one which receives little or no attention in the regular course of lectures, or in the majority of your text-books. Though a subject of such importance to medical men, as well as to humanity in general, it is more fully discussed by quacks than educated physicians. This city, and every other large city, has a prolific growth of these people, and they find hosts of victims, whom they return to us as worse than ruined by their pernicious advice, and their still more pernicious treatment. There is so much surface modesty nowadays that it is hardly considered proper to make a study of the functions of generation, or a study of the pathological conditions, or the habits which transmit disease to generations yet to come. The principal remedy for this is for medical men to become as familiar with the pathology and treatment of the varied forms of spermatorrhœa and impotence, and the long series of constitutional troubles which accompany them, as they are with the treatment of a diseased lung or a broken leg, and to disseminate such knowledge of the consequences of these diseases, the result of uneducated and uncontrolled desire, among their patients and friends capable of appreciating their motives and their advice.

But before entering upon a study of the morbid conditions referred to, let me briefly refer to the normal physiological condition of the organs involved, so that you may more readily understand the pathological condition.

The seminal fluid secreted by the testicles passes through the vas deferens to the vesiculæ seminales at the base of the bladder, where it is stored up for future use. From these sacs it passes out through the ejaculatory ducts into the urethra, becoming mixed, as it passes through the latter, with the secretions of the prostate and Cowper glands. I am aware that some think the office of the vesiculæ seminales is *not* to retain a reserve quantity of spermatic fluid; but a storehouse *is* necessary for its reception, and as there is no other sac anywhere near which would answer the purpose, I conclude that their office is just as I stated. There is no proof whatever that the surplus stock of seminal fluid is absorbed to enrich the blood. The statement is guess-work. In the normal condition the secretion is constant, and as soon as the vesiculæ seminales become filled, some of it must be thrown out by nocturnal emissions, to make room for the newly manufactured fluid coming from the testicles. Where sexual intercourse is not indulged in, emissions may occur as often as once a week, though a majority of healthy men do not have them more than once in two or three weeks. In the healthy state emissions never occur during waking hours—only at night; and the erections and pleasurable sensations are of the same character as accompany sexual intercourse. The sensation which accompanies the orgasm is produced by congestion and distention of the veru montanum in the prostate portion of the urethra.

Emissions are not confined to the male—they are of common occurrence in the female; the secretion which is poured out at the time of the orgasm comes from the glands of Bartholini, situated on each side of the commencement of the vagina, external to its walls. These emissions occur at night, in connection with lascivious dreams, in precisely the same manner that they arise in the male.

The erection of the penis, which precedes the emission of seminal fluid, is due primarily to a distention of the erectile tissue of the organ with blood. If you examine this erectile tissue you will see that it resembles somewhat the structure of an ordinary sponge; that it consists of innumerable spaces, irregular in form and size, with elastic walls capable of great distention. In these cavities are found thin-walled blood vessels, also capable of an immense increase in size. These vessicular channels, as in other parts of the body, are under the control of the sympathetic nervous system, and through the sympathetic nervous system their calibre is regulated. Emotions of various kinds, which I need not enumerate here, cause the vessels of the erectile tissue to dilate, a large quantity of blood is forced into them, until the whole organ is distended to twice its ordinary size. The distension of the blood vessels is similar to that which occurs in blushing; a thought will cause the capillaries of the face to dilate, and the skin to become red; an emotion of a contrary kind will produce sudden contraction of the capillaries, and consequent pallor. Both occurrences are completely beyond the control of the will. So it is with erections. The will has little or nothing to do with them; one variety of emotion will excite them, and another destroy them, and this is an important fact for you to remember in the treatment of impotence.

The permanency of the erection depends: 1st, on an increased afflux of blood to the part; and 2d, a certain amount of obstruction to the return circulation from the organ, produced by contraction of the muscular fibres of the accelerator urine and erector penis. Many physiologists deny that there is any obstruction to the return circulation; that the erection is maintained simply by the increased quantity of blood sent to the organ. Well, I don't agree with them. It is a fact that the muscles mentioned are contracted during erections. From their situation and attachments it is impossible for them to contract without obstructing more or less the blood *coming from* the penis. Besides this there is a physiological reason for the stoppage of the current outwards. The penis, as you are aware, sometimes gets into a tight place, and if there were no impediment to the return circulation, the blood would be forced out during intromission, and a consequent failure of the erection would result. I don't mean to say that the obstruction is complete by any means, but there is enough to make the organ rigid, no matter how much compression by natural means is brought to bear on it.

We know very little about the elements which govern the erectile power. Gall, who developed phrenology, located it in the cerebellum. When this portion of the brain is removed from the cock before placing him among his family of hens, he attempts to perform his duties as of old, but is unable to steady himself for the act, and finally goes off disgusted. Here you see there is still *desire*, but the bird is rendered incapable because he cannot co-ordinate his movements. Removal of the cerebellum has caused atrophy of the genitals, and irritation of the organ from disease has been followed by satyriasis in the male, and nymphomania in the female. There can be little question, therefore, that the cerebellum has more or less influence over the function of generation; what its special work is remains yet to be determined.

There is a nerve-center in the cord opposite the lumbar vertebræ called the genito-spinal center, from which nerves can be traced which supply the genital organs. An irritation applied at this point will produce erections of the penis and contraction of the bladder, and in the female contraction of the uterus.

*Masturbation.*—Now, as to the causes of spermatorrhœa or impotence. They are very numerous, but masturbation takes the first place in the majority of cases. Masturbation is an universal vice in civilized countries among both sexes. Savage races are free from it. They are under no obligation, moral or otherwise, to abstain from the natural gratification of their passions—are not compelled by social laws to govern their sexual appetites. There is no reason, therefore, why they should resort to irregular means of relief.

Masturbation has been noticed among lower animals, as dogs, rats, etc., but I am inclined to think it exceedingly rare, and that the animal so indulging must have been taught the act by some depraved animal of our own species.

Children begin the habit at a very early age. Instances are on record of babes at the breast doing it; but I should want ocular proof of them before believing. It is no uncommon thing, however, for children to commence masturbation at the age of five years. From that period to the age of sixteen might be called the masturbation age, though the vice may be indulged in at much later periods of life.

There are certain gymnastic exercises which are provocative of masturbation, and which should be prohibited until manhood is reached. These exercises, too, are common in all our school gymnasiums, as well as public gymnasiums. My attention was first directed to this important fact a few years ago by the history which a confirmed masturbator gave me of his first experience. When a lad of eight he commenced exercising in a school gymnasium. One day, while using the swinging pole, sustaining the whole weight of his body by his hands, he felt a peculiar sensation in his genital organs, which became so intense that he was compelled to sit down. Every time he resumed the exercise the same pleasurable sensations returned. They led him to a closer examination of his genital organs, and subsequently to new methods of increasing the excitement, until he became a confirmed masturbator. Since then I have had a somewhat similar history from five other persons, one a female, confirming the opinion which I now hold, viz: that all exercises, such as climbing and sliding down poles, swinging from rings or ropes—the body being supported by the hands alone—should be dispensed with until the age of twenty or thereabouts; then there is less danger of determination of blood to the genitals as a consequence of these exercises.

When the vice of masturbation is fully developed it is rarely, if ever, discontinued before the age of twenty, and it is often continued to a later period. About that time the effects of the sin manifest themselves so plainly that the fears are aroused, and an attempt at reformation made—an attempt which generally succeeds in men not entirely given over to the dominion of ungovernable and beastly sensuality. A few years of masturbation leave characteristic local marks on the genitals, even though the rest of the body give the appearance of health, which is rarely the case. The local lesions are sufficient for a diagnosis, even when there is no history of the case obtainable. The penis is much thinner than normal elongated sometimes, with a glans much larger than the body of the organ. This is due to the gravitation of the blood, which fills and distends the easily dilated vessels of the glans in the pendant position. The veins of the integumental covering are dilated and varicose. The penis is often bent laterally, generally to the left side, a condition due to partial paralysis of the opposite side. I have, in such cases, found the opposite or convex side to be deficient in sensibility. A like condition may be present in persons who have indulged too much in venereal excesses. The testicles are smaller and softer than natural, and hang low down on the relaxed scrotum.

The mucous membrane of the urethra is congested throughout; but the most marked changes are noticed in the prostatic portion of the urethra. In this condition the mucous membrane was very much thickened and congested. The veru montanum is increased to twice its ordinary size, being often so large as to afford an obstacle to the passage of the sound. The whole mucous surface of the prostate presents very much the same appearance as granular lids do in long standing cases, with the difference that the color is much darker. If you examine the orifices of the prostatic ducts which lie on each side of the veru montanum, you will find that they are much larger than normal, darker colored than the rest of the mucous membrane, divested of epithelium, like the other parts, and containing considerable muco-purulent exudation. Occasionally ulceration exists at the orifices of these ducts. The mouths of the ejaculatory ducts, which open near the edge of the *sinus pecularis*, in front of the veru montanum, present similar changes. The prostate gland is enlarged, and is often tender on pressure. Pressure on the gland through the rectum will generally be followed in a few moments by the appearance of an opaque fluid at the meatus, which is partly prostatic secretion and mucus, containing epithelial cells. You will also find that these patients have a frequent desire to micturate, and micturition is rarely accomplished without some sense of discomfort at the neck of the bladder, or rather at the prostatic portion of the urethra, where it is irritated by the salts of the urine passing over it. The passage of a sound is followed, in many instances, by a discharge of a clear viscid mucus. This viscid mucus also occurs in large quantities after excessive venereal excitement.

If the patient has been frightened out of his bad habits, or has ceased them from any cause, he will, in a short time thereafter, be troubled with emissions of seminal fluid at night, accompanied by dreams, sometimes with erections, but oftener without them. These emissions vary in number from two to four and five per week, and they may occur during the daytime from slight sexual excitement. Such a condition is known as spermatorrhœa, though the term, in its strictest application, means a flow of seminal fluid, without erections, desire or pleasurable sensations—an extremely rare condition. When the emissions appear the patient's fears are increased. He believes that he is ruined beyond hope of recovery. If he attempt sexual intercourse, he is sure to fail; and that is about the only good thing which results from the habit of masturbation, for during these years of inordinate sexual excitement he will often have endeavored to cohabit with girls of his acquaintance, whose ruin would be accomplished were it not for the weakness and nervousness which make him impotent. The majority of masturbators are for a time impotent, and consequently harmless.

In connecion with emissions of seminal fluid in patients who have masturbated or indulged greatly in sexual intercourse, there is often a discharge of opaque mucus which occurs independently of venereal excitement, and which is considered by the patient to consist of seminal fluid. If you examine it under the microscope, you will find it made up of pus and epithelial cells; there are no spermatozoa in it. Never call this condition spermatorrhœa.

It is generally supposed that every masturbator carries his sign out, and that the face is a never-failing "tell-tale." Such, gentlemen, is not the case. The majority of masturbators don't show any more than married men do the results of free sexual intercourse. It is only when the vice has been indulged in to excess for a long period of years, when it has destroyed all the finer instincts of manhood, that you notice the pale expressionless face, with sunken eyes that seldom meet yours, but steal sidelong glances when your attention is drawn another way. You will find in such instances also a cold, clammy skin, the superficial veins of the integument dilated, the tongue coated, breath bad, and bowels always constipated. You will find them cowardly, easily startled, sleepless, inanimate, forgetful, stupid, often troubled with epilepsy, and occasionally with the mind on the verge of insanity—mere animals in everything, in desire and in acts.

*Mental Emotion as a Cause of Impotence.*—The vast influence of the mind over the organic functions cannot be over-estimated. As medical men we see it everywhere in the course of our daily practice. We know from what we have seen that a "strong imagination will create what it imagines" with very little trouble. We see it among the manifestation of hysteria in a very marked degree. I remember, when an interne at Bellevue, an hysterical woman who was placed near a patient suffering from mammitis. The morning after her admission she complained of a pain in her breast, and on examining it I

found it swollen and much larger than the one on the corresponding side; yet there was no fever nor any other sign of inflammation. I have seen another in the same way get an attack of pseudo-peritonitis, and still another develop all the symptoms of a real inflammatory softening of the brain, all copied from other patients who were really afflicted with the diseases in question.

Now, there are no organs in the body so readily influenced by mental emotions as the genital organs, and mental emotion is one of the commonest causes of impotence in existence. Impotence of this nature may occur in married or single men in comparative health, as well as in those who have masturbated to excess. In the latter it is more apt to take place, but you will find very many cases where there is no other assignable cause but mental emotion. Troubles in business, anxiety about the health, family jars, a fit of indigestion, a disagreeable remark from one of the interested parties, a leucorrhœal discharge with a fetid odor—in fact, any uncomfortable feeling whatever which takes the mind away from the act, will bring on impotence; and, when one failure occurs, no matter how trivial the cause, others are sure to follow. Fear is an essential element in many cases—fear that the act cannot be accomplished; it is this fear which produces impotence in many who have masturbated. They read in advertisements and elsewhere of their loss of manhood, and the very first attempt at intercourse fails, even when there is really no loss of power. Temporary impotence has been produced in a healthy man by a friend's recital of his own surprising failure. The thought of the accident which befel the friend occurred at the wrong time and he too failed. I had a patient last year troubled with gleet; if he noticed the discharge before retiring it affected his mind so much as to render him incapable. Another that I know is only impotent with his own wife; with strange women he has no trouble whatever. Here, of course, there must have been an absence of that magnetism which should exist between man and wife, and perhaps an uncontrollable aversion added to it, both working together to induce the failure. Roland speaks of a man who just had connection for the first time with a woman fully dressed, and who continued the intercourse under similar circumstances for some months. Subsequently he married, but found, much to his astonishment, that he was unable to begin or even to complete the act. The previous intercourse with a woman in full dress had disturbed his equilibrium, and he could only perform his part with the first surroundings. I believe that he was fully able to conquer this feeling completely. I might go on with innumerable instances of such cases of impotence, but our time will not admit of it; neither do I think it necessary. But I do wish you to be impressed with the idea that emotions cause impotence in most patients, and that such impotence is always amenable to treatment, and can, with rare exceptions, be recovered from.

*Malformation of the Genitals.*—A week ago I saw a patient in Charity Hospital who had a large cicatrix near the corona glandis, involving the integument. When erection took place the glans became bent on the body almost at right angles. He suffered considerable pain at such times, and was unable to effect intromission. An appropriate incision cured him. Bending of the organ on itself from the contraction of cicatricial tissue is not uncommon, and it can generally be relieved by the knife.

Tumors of the penis also cause impotence, the enlargement preventing intromission.

Cases of double penis involve impotence, unless vaginæ can be found to match them.—*Medical Record.*

